# Tackling waste in publishing through portable peer review

**DOI:** 10.1186/s12915-018-0619-z

**Published:** 2018-12-17

**Authors:** Graham P. Bell, Mirna Kvajo

**Affiliations:** Springer Nature, New York, USA

## To speed up the peer review process and decrease waste in publishing, *BMC Biology* formalizes and extends its portable review policy

High quality and timely assessment of manuscripts are the cornerstones of peer review, which plays a central role in validating research results and advancing scientific discovery. However, the evolution of the publishing and research landscape is bringing challenges to the peer review process: reviewers struggle with the burden of growing numbers of papers to assess, finding peer reviewers for manuscripts is overall becoming harder [1], and authors worry about the time it takes for their papers to reach the community. Peer reviewed papers that have been rejected, often due to the interest threshold of a journal, require the engagement of new sets of reviewers upon submission to the next journal, increasing the overall effort and prolonging the publication process. These concerns have spurred explorations of ways to improve peer review [2], including measures aimed at increasing the efficiency of the process. One such idea is portable peer review: the concept that reviews obtained at one journal can be transferred and re-used by others [3].

*BMC Biology* has for many years supported portable peer review practices [4]. As part of BMC we offer easy and efficient transfer of manuscripts to other journals within our publishing group, and we have recently also joined the NPRC alliance, a consortium of more than 60 journals committed to improving peer review in neuroscience. To further extend the benefits of portable peer review we are now introducing a new Transfers and Portable Reviews Policy, with which we offer to share reviews from *BMC Biology* (along with reviewers’ identities, subject to their approval) with any journal of the authors’ choice, including those outside of BMC and Springer Nature.

For the past 5 years, *BMC Biology* has also considered papers reviewed at other journals on the basis of the existing referee reports, with the support of our Editorial Board. In this way, we have been able to publish many impactful and robust studies while reducing time to publication and the effort of reviewers. We see that these practices benefitted authors, reviewers and ultimately research, and to further encourage portable peer review at *BMC Biology*, we are now formalizing them into a policy. We want to let potential authors know that they are welcome to come to us with their reviews, and we’ll chart together the path forward for the papers. Authors wishing to take up this route can simply email us to enquire about the suitability of a manuscript, with information about previous journal’s identity and all reviews. We then typically ask the previous journal to share with us the reviews and reviewers’ identities, and—subject to agreement from all parties—original reviewers may be consulted if deemed appropriate.

With these changes we want to support a pragmatic and streamlined peer review process that will allow robust science to thrive, while minimizing the challenges ailing the current process.
